# A mixed methods feasibility study of a ketogenic diet as treatment for Parkinson’s disease

**DOI:** 10.3389/fnut.2025.1601446

**Published:** 2025-07-23

**Authors:** Kate Worster, Dana Colgan, Alexandra Vita, Christine McClure, Lita Buttolph, Romilly Hodges, Angela Senders, Andy Erlandsen, Fawzy Elbarbry, Heather Zwickey

**Affiliations:** ^1^Helfgott Research Institute, National University of Natural Medicine, Portland, OR, United States; ^2^Department of Neurology, School of Medicine, Oregon Health Science University, Portland, OR, United States; ^3^Department of Food Science and Human Nutrition, Colorado State University, Fort Collins, CO, United States; ^4^Department of Neurology, Oregon Health & Science University, Portland, OR, United States; ^5^Sonoran University, Tempe, AZ, United States; ^6^School of Pharmacy, Pacific University, Hillsboro, OR, United States

**Keywords:** ketogenic diet, Parkinson’s disease, patient-initiated, gut microbiome, inflammation, glucose

## Abstract

**Objective:**

People with Parkinson’s disease (PD) have been shown to benefit from a ketogenic diet (KD). However, evidence suggests the traditional KD’s high dairy consumption may exacerbate PD symptoms. This patient-initiated study assessed the feasibility and acceptability of a novel ketogenic diet limiting dairy products in patients with PD. Quality of life and functional movements were also evaluated.

**Methods:**

Twelve people with PD followed a modified, low dairy KD for 12 weeks. We provided support and nutritional education to assist with adherence. Subjects recorded daily food diaries, from which total macronutrients were calculated. Every 4 weeks blood (complete blood count (CBC), lipid panel, vitamin D, beta-hydroxybutyrate, electrolytes), urinalysis (calcium, creatinine), vitals, height, weight, quality of life [Parkinson’s Disease Questionnaire-39 (PDQ-39)] and functional movement assessments [Unified Parkinson’s Disease Rating Scale (UPDRS), Freezing of Gait, mini-Balance Evaluation Systems Test (mini-BEST), 360° Turn] were collected.

**Results:**

All subjects completed the study and 75% recorded at least three-quarters of their daily food diary entries. Average macronutrient levels (70% fat, 18% protein, 5% net carbohydrate) and beta-hydroxybutyrate levels (*p* < 0.005) confirmed nutritional ketosis was maintained. Clinical improvements were found in total UPDRS, UPDRS Part III, miniBEST, Freezing of Gait, and quality of life. All participants lost weight; 58% reported no change in constipation and 8% reported improvement.

**Conclusion:**

This exploratory study deemed the modified ketogenic diet feasible and acceptable. Findings suggest a low dairy KD may provide similar benefits to a traditional KD for those with PD, while reducing potential risks associated with consuming higher amounts of animal dairy products.

## Introduction

The prevalence of Parkinson’s disease (PD) has doubled in the last 25 years and is estimated to affect 8.5 million individuals globally (1). It is the second most common neurodegenerative disease after Alzheimer’s disease (2). Although the exact pathogenesis is unclear, PD is characterized by misfolded alpha-synuclein protein accumulation leading to neuronal death, particularly in the substantia nigra’s dopaminergic neurons (3). Exacerbating this neurodegeneration are the involvement of pro-oxidant pathways, decreased mitochondrial function (4–6), inflammatory responses associated with elevated proinflammatory cytokines, increased levels of circulating insulin and glucose (7), and inflammasome alteration of the gut microbiota that may be both activated by and promote further alpha-synuclein aggregations (3). This energy dysregulation seen in PD may partially explain why the switch in cellular energy substrate from glucose to triglycerides associated with a ketogenic diet (KD) may be neuroprotective and potentially mitigate symptoms (8).

**Figure 1:**
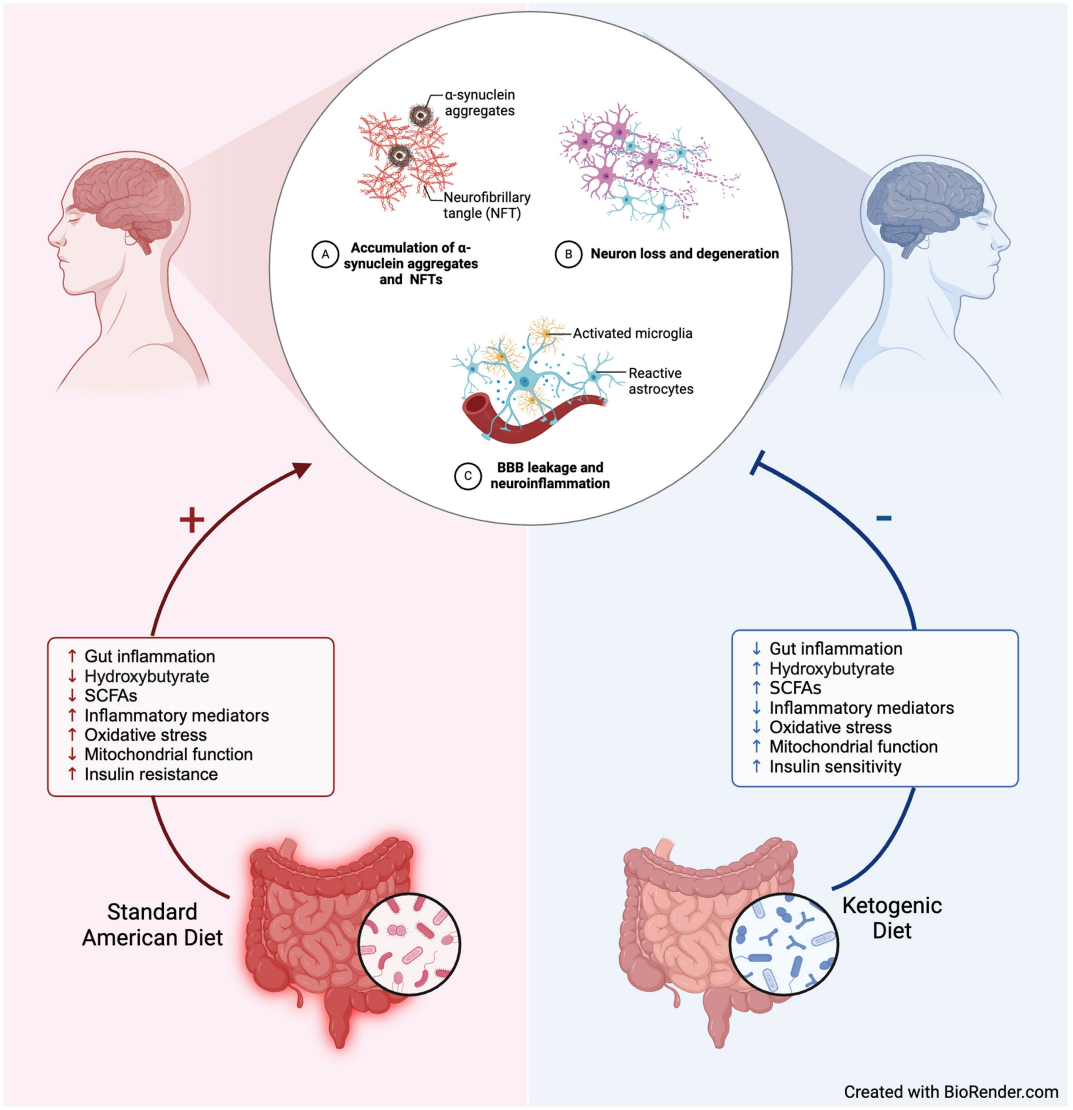
Hypothesized mechanisms underlying neuroprotective benefits of ketogenic diet for Parkinson’s. Created with BioRender.com.

During a prolonged restriction or starvation of carbohydrate intake, ketone bodies become the primary energy source of adenosine triphosphate (ATP) for cells, including neurons. Hepatically produced beta-hydroxybutyrate (BHB) and acetoacetate are the two primary ketones of nutritional ketosis; with BHB having the neuroprotective benefit of crossing the blood–brain barrier and preventing hippocampal neuron death (4). Other neuro-beneficial effects of a predominantly ketone-predominant metabolism are increased central nervous system (CNS) adenosine, increased gamma- aminobutyric acid (GABA) levels, and decreased glutamate levels, all of which directly affect ion channels, increase mitochondrial ATP production, and have anti-inflammatory and antioxidant effects (Figure 1) (9).

Traditionally, a KD includes significant portions of animal dairy foods as a fat source. While the relationship between dairy, gut microbiota, and PD is not fully elucidated, we propose dairy is an important factor to consider when designing a KD for these patients due to studies showing high dairy product consumption, particularly low-fat, is associated with an increased risk for PD. (10, 11).

Although there are pharmaceutical treatments for PD, many patients feel overwhelmed by managing polypharmacy over the disease course and may experience adverse events, functional decline, cognitive impairment, falls, and other negative health outcomes (12). Therefore, patients are increasingly advocating for non-pharmacological treatment options that may be used alongside other interventions. This study was initiated by a patient support group whose members were investigating additional options for managing disease progression and discovered two articles about the neuroprotective effects of a KD (5, 8). The group collectively expressed interest to their physician and local researchers to investigate whether a KD could positively impact quality of life and help control certain aspects of their disease process. The novel KD designed for this study has an important distinction of limiting dairy due to evidence linking high dairy intake with increased PD risk. The primary aim of this 12-week, patient-initiated, mixed-methods, exploratory study was to evaluate the feasibility and acceptability of an innovative, modified KD. A secondary aim included assessing the potential impact of this diet on the quality of life and functional movements in patients with PD.

## Materials and methods

### Ethics and consent

The study protocol was approved by the IRB (IRB#00000677) at Legacy Health System, and the IRB at the National University of Natural Medicine (NUNM) deferred to Legacy’s IRB. All study participants signed written informed consent forms to participate in the study. To ensure the confidentiality of patient information, patients’ names and addresses, such as phone numbers, were not recorded during data collection. The entire study process followed the relevant guidelines and regulations of the Declaration of Helsinki.

### Recruitment

Participants were recruited through the study physician and patient support group at Legacy Health System in Portland, Oregon.

### Inclusion criteria

Participant inclusion criteria included a PD diagnosis per the British Brain Bank criteria (13), disability Hoehn-Yahr stage 2 or 3 (14), and stable dose of PD medication for a minimum of 3 months before study enrollment and for the duration of the study.

### Exclusion criteria

Participants were excluded if they had significant heart disease, including uncontrolled significant hyperlipidemia, coronary artery disease, and congestive heart failure; evidence of diabetes; cancer other than basal cell skin cancers; kidney stones or gallstones; genetic conditions regarding metabolism; and not currently pregnant or planning pregnancy during the study.

### Study design overview

Before the trial, participants were interviewed about their reasons for participating in the study, any concerns, their ability to track macronutrient intake, and any baseline gastrointestinal issues. This data was analyzed qualitatively. Biomarker and anthropometric data were collected from all participants at weeks 0, 4, 8, and 12. Measures included blood pressure, height, weight, complete blood count (CBC), lipid panel, multi-chemistry panel (i.e., albumin, total protein, AST, ALT, BUN, creatinine, glucose), vitamin D, electrolytes (i.e., serum bicarbonate, calcium, zinc, selenium, magnesium, and phosphate), serum BHB, urinalysis, urine calcium and creatinine. Subjects were provided with a KetoCoach™ blood ketone meter kit to measure BHB at home and given instruction and practice on use. Quality of life and functional movements were also assessed at weeks 0, 4, 8, and 12 via self-report and behavioral assessments. Quality of life measures were used to determine the acceptability of the dietary intervention.

### Nutritional intervention

Participants were instructed to eat a KD (goal 80% kcal fat, 15% kcal protein, and 5% kcal net carbohydrate) for 12 weeks. All participants and their primary food-preparer attended weekly visits at the National University of Natural Medicine (NUNM) Helfgott Research Institute in Portland, Oregon to support and assess dietary adherence. Educational support began 1 week prior to starting the KD and included how to stock a pantry and refrigerator for the diet. Ongoing educational support included KD details with highlighted foods, recipes, and menus each week. The Parkinson’s version of the KD was modified to recommend minimal dairy, plant-based foods for fiber, and the inclusion of essential micronutrients. Participants recorded their daily food intake with the phone app, MyFitnessPal.

### Primary outcomes

In this exploratory study, primary outcomes were feasibility and acceptability data. Predetermined satisfactory outcomes of feasibility included: (i) less than a 30% withdrawal rate; and (ii) evidence of diet adherence. Diet adherence was evidenced by participants recording at least 75% of total macronutrient tracking diaries, participants’ overall mean macronutrient levels recorded as approximately 80% fat, 15% protein, and 5% net carbohydrate, and an indication of nutritional ketosis via a significant increase of beta hydroxybutyrate throughout the intervention. Predetermined satisfactory outcomes of acceptability included: (i) maintaining quality of life, evidenced by quality of life not decreasing more than 25%; and (ii) no significant worsening of constipation throughout the intervention. Further, acceptability was explored via participants’ baseline concerns regarding a ketogenic diet and reasons for participation in the study. Predetermined criteria for satisfactory levels of safety included: (i) weight loss no greater than 10% of baseline weight; and (ii) no significant changes in measures of blood cholesterol, vitamin D, kidney function, electrolytes, or immune cells throughout the intervention.

### Secondary outcomes

PD quality of life and functional movement assessments were collected at baseline and every 4 weeks, including Unified PD Rating Scale (UPDRS) (15), PD Questionnaire-39 (PDQ-39) (16), Freezing of Gait (FOG) (17), miniBEST (18), and 360° Turn (19).

### Quantitative data analysis

Descriptive statistics (i.e., mean, standard deviation, percent change) were calculated in Microsoft® Excel®. Non-parametric t-tests, with an alpha level of 0.05, using the built-in Matlab (R2024a) function *ranksum* were conducted to assess for changes in blood cholesterol markers, kidney function, immune cells, electrolytes, and vitamin D. The percentage of participants who reported or demonstrated a minimal clinical important difference (MCID) was identified for UPDRS-Total, UPDRS III, UPDRS IV, PDQ-39, FOG, miniBEST, and 360° Turn.

### Qualitative data analysis

A trained researcher conducted individual interviews using a semi-structured interview guide. Participants were interviewed alone or accompanied by a family member and were invited to ask questions and provide additional details or feedback throughout the interview. Analyses were conducted using conventional content analysis in which coding categories were derived directly from the text data (20). This method systematically examined material and obtained a condensed description of content (20).

## Results

### Participants

Twelve participants were enrolled in the study and all completed the study. Participants completed the dietary intervention in cohorts of four. Eight participants completed the qualitative, pre-study interview. Table 1 provides the participants’ socio-demographics and characteristics. Two of the eight individuals interviewed (17%) reported previously adhering to a similar diet (e.g., Atkins, Paleo).

**Table 1:** Sociodemographic of the 12 study participants.

Characteristic	Participants
Number (sex)	6 (female), 6 (male)
Mean (SD) age, yr	64 (5.80)
Ethnicity	11 Caucasian (92%), 1 Chinese (8%)
Mean (SD) time since diagnosis, yr	4.25 (3.86)

### Feasibility

**Figure 2:**
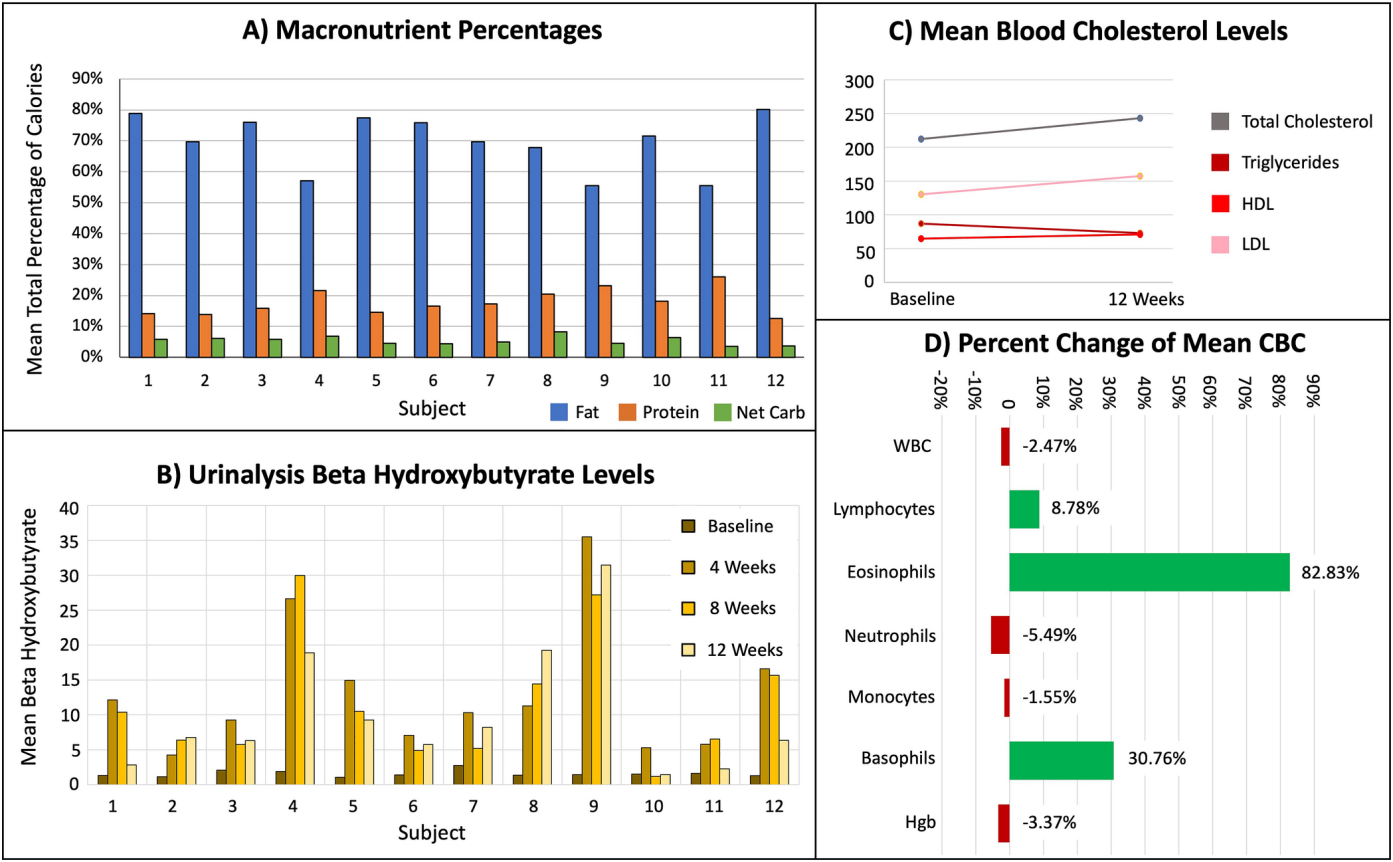
Results from dietary tracking, urinalysis, and blood tests, indicating subjects achieved nutritional ketosis. **(A)** Mean total percentage of macronutrients. **(B)** Mean BHB levels for every 4 weeks of the protocol. **(C)** Mean baseline and week 12 serum levels for total cholesterol, triglycerides, HDL, and LDL. **(D)** Percent change of mean white blood cell (WBC), immune cells (lymphocytes, eosinophils, neutrophils, monocytes, basophils), and hemoglobin (Hgb).

Seventy-five percent of participants entered data for at least 75% of the total macronutrient tracking diaries. Recorded mean macronutrient levels during the study consisted of 70% fat, 18% protein, and 5% net carbohydrate (Figure 2A). These mean macronutrient percentages are derived from cohort mean calculations are not expected to total 100%, as they represent the average of individual percentages rather than a proportion of a single pooled value. Urinalysis BHB levels indicated all participants were in nutritional ketosis (KetoCoach™ kit reference range 2.0–7.0 mM/L) by week 4, evidenced by a statistically significant difference in BHB levels from baseline to Week 4 (*p* < 0.01), baseline to Week 8 (*p* < 0.01), and baseline to Week 12 (*p* < 0.01; Figure 2B). Each of these measures met our pre-defined feasibility criteria.

### Acceptability

**Figure 3:**
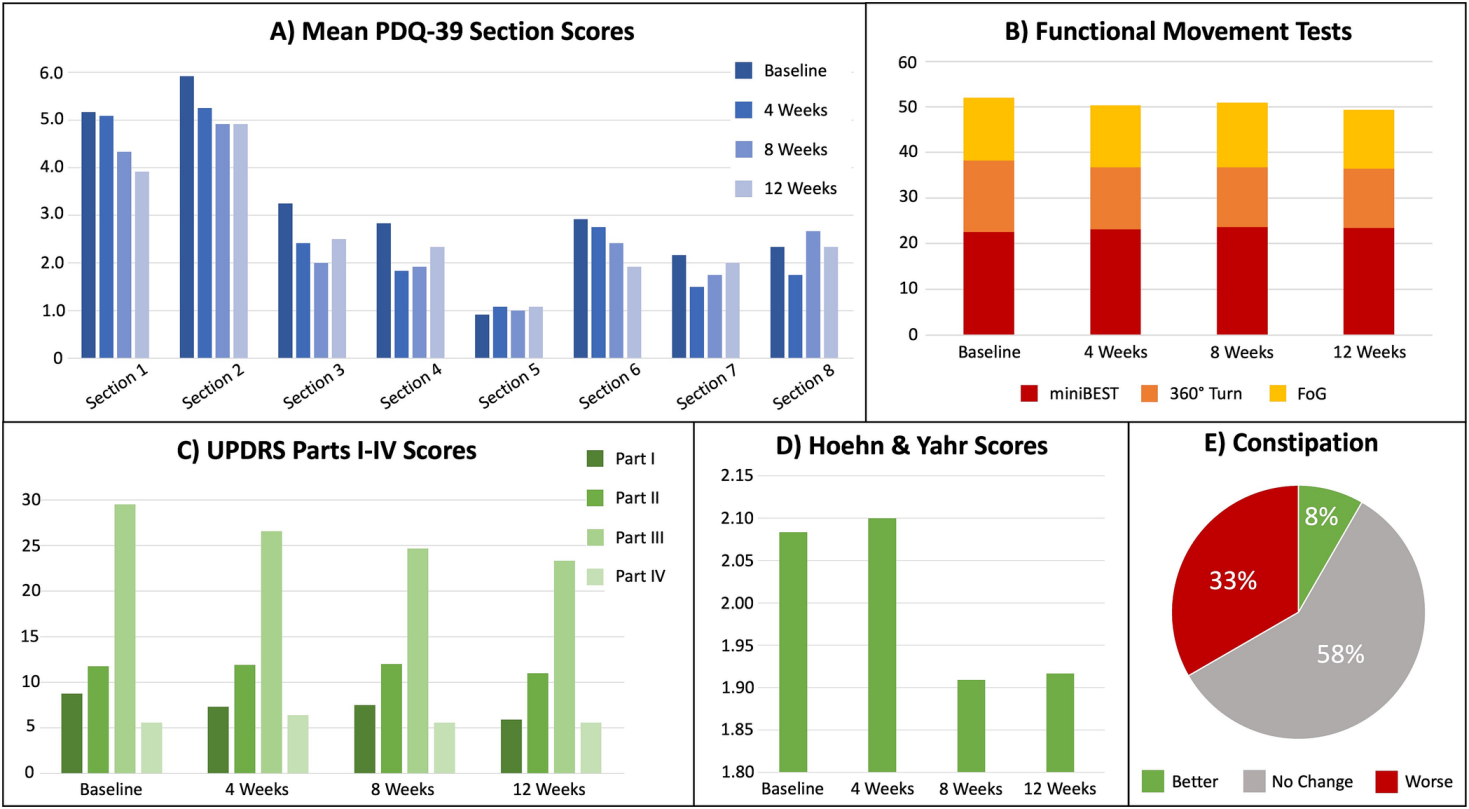
Results for metrics of the cohort’s quality of life, functional movement tests, and severity of Parkinson’s disease. **(A)** Mean PDQ-39 scores (higher = worse) by section, where Section 1 = mobility, Section 2 = activities of daily living, Section 3 = emotional well-being, Section 4 = stigma, Section 5 = social support, Section 6 = cognition, Section 7 = communication, and Section 8 = bodily discomfort. **(B)** Mean functional movement test scores for miniBEST, 360° Turn, and FoG, where higher = worse. **(C)** Unified Parkinson’s Disease Rating Scale (UPDRS) mean scores for each section, where higher = worse. **(D)** Mean Hoehn and Yahr scores from UPDRS, where higher = worse. **(E)** Percentage of self-reported constipation severity (UPDRS section 1.11).

Quality of Life- PDQ-39 scale scores indicated all participants maintained or improved quality of life (Table 2). Further, 58% of the sample reported no change in constipation (Figure 3E). One participant reported improved constipation and four reported a slight worsening. Qualitative data from the baseline participant interviews explored participants’ acceptability of a KD. Reasons for participating in the study included: (i) perception that the KD might assist in maintaining active lifestyles and slowing disease progression (75%); (ii) importance of contributing to scientific knowledge (50%); (iii) curiosity about the KD and possible effects on PD (37%); and (iv) recommendations by family members to enroll in the study (37%). Further, all participants communicated a willingness to track macronutrients.

**Table 2:** Mean (standard deviation) and minimally clinically important difference (MCID) for quality of life and functional movement assessments.

				MCID	
Assessment (*n* = 12)	Baseline	Week 12	Improved	Same	Declined
360° Turn	15.64 (9.96)	12.99 (1.56)	N/A	N/A	N/A
miniBEST	22.58 (3.82)	23.42 (1.93)	25%	58%	17%
FoG	13.83 (15.10)	13.00 (11.01)	25%	25%	50%
*Unified Parkinson’s disease rating scale*					
Total UPDRS			60%	15%	25%
UPDRS Part I	8.75 (5.05)	5.92 (4.78)	N/A	N/A	N/A
UPDRS Part II	11.75 (6.14)	11.00 (8.58)	N/A	N/A	N/A
UPDRS Part III (Mobility exam subset)	29.50 (12.89)	23.33 (5.97)	50%	25%	25%
UPDRS Part IV	5.58 (4.01)	5.58 (3.00)	0%	100%	0%
Hoehn Yahr (section 1.11)	2.08 (0.51)	1.92 (0.29)	N/A	N/A	N/A
*Quality of Life – PDQ-39*					
Section 1 - Mobility	5.17 (8.36)	3.92 (4.56)	25%	50%	25%
Section 2 - ADLs	5.92 (6.13)	4.92 (4.38)	25%	50%	25%
Section 3 - Emotional well-being	3.25 (4.20)	2.50 (2.78)	42%	58%	0%
Section 4 - Stigma	2.83 (3.01)	2.33 (1.97)	16%	66%	8%
Section 5 - Social support	0.92 (2.11)	1.08 (2.07)	0%	100%	0%
Section 6 - Cognition	2.92 (1.68)	1.92 (1.93)	42%	58%	0%
Section 7 - Communication	2.17 (2.08)	2.00 (2.37)	8%	83%	8%
Section 8 - Bodily discomfort	2.33 (2.10)	2.33 (2.27)	17%	58%	25%

N/A indicates MCID values were not available in the current literature.

Approximately 40% of the sample reported no concerns and described being very optimistic/enthusiastic about following a KD. In contrast, about 60% of the sample described various concerns of starting a KD, including the possibility of increased constipation, lack of energy, increased cholesterol, carbohydrate cravings, and undesired weight loss. Other concerns included a possible lack of motivation to adhere to the diet, contra-indications with medications, unknown duration of maintaining ketosis, and adherence to the diet when traveling. Interestingly, almost 90% of participants interviewed reported baseline constipation. Five of the eight reported use of a stool softener (e.g., fiber, magnesium, Metamucil™, gobo root, unidentified stool softener) to reduce constipation. Gas and bloating at baseline were reported by 37% of the sample interviewed. Three participants reported having a daily bowel movement and four reported one bowel movement every other day.

### Safety

All participants lost weight during the study, ranging from 1.60 to 7.48% of their baseline. At baseline, the cohort’s mean total cholesterol 212 (±37.71) and LDL 130.17 (±24.67) were above normal. All other baseline peripheral blood safety measures were within normal limits. From baseline to week 12, there were no statistically significant differences in immune cell percentages, lipid panel (i.e., total cholesterol, HLD, LDL, triglycerides), multi-chemistry panel (i.e., albumin, total protein, AST, ALT, BUN, creatinine, glucose), vitamin D, electrolytes (i.e., serum bicarbonate, calcium, zinc, selenium, magnesium, phosphate), urine calcium, and urine creatinine (Table 3).

**Table 3:** Mean (standard deviation) and non-parametric *t*-test for cohort’s serum and urinalysis laboratories collected at baseline and week 12.

Variable (*n* = 12)	Baseline	Week 12	*p*-value
Weight (lb)	186.37 (38.71)	177.73 (37.67)	*p* = 0.4703
Lipid panel			
Total Cholesterol (mg/dL)	212.17 (37.71)^†‡^	243.08 (53.28)^†‡^	*p* = 0.1749
Triglycerides (mg/dL)	87.25 (29.22)	73.25 (23.03)	*p* = 0.2366
LDL (mg/dL)	130.17 (24.67)^†‡^	157.42 (47.21)^†‡^	*p* = 0.2717
HDL (mg/dL)	64.75 (14.59)	71.08 (13.37)	*p* = 0.1332
Serum analysis			
Beta-hydroxybutyrate	*1.57 (0.46)*	*9.90 (8.89)*	*p = 1.7432 E-05*
Vitamin D	35.50 (14.50)	38.67 (22.56)	*p* = 0.9309
HgB	14.21 (1.14)	13.73 (1.19)^†^	*p* = 0.3105
White blood cells (%)	5.24 (0.76)	5.09 (1.39)	*p* = 0.5246
Lymphocytes (%)	31.58 (7.19)	32.76 (4.18)	*p* = 0.7491
Eosinophils (%)	3.25 (2.28)^†^	4.93 (3.94)	*p* = 0.4341
Monocytes (%)	9.73 (1.42)	9.50 (1.37)	*p* = 0.7221
Neutrophils (%)	55 (7.11)	52.21 (5.06)	*p* = 0.2705
Basophils (%)	0.44 (0.18)	0.60 (0.27)	*p* = 0.1832
CBC			
Albumin	4.30 (0.22)	4.38 (0.20)	*p* = 0.2903
Total protein	6.92 (0.38)	7.00 (0.33)	*p* = 0.4853
AST	22.67 (6.30)	27.50 (11.96)	*p* = 0.1225
ALT	20.58 (14.37)	30.58 (15.47)	*p* = 0.0525
BUN	14.75 (3.52)	15.17 (4.49)	*p* = 0.8391
Creatine	0.89 (0.15)	0.81 (0.16)	p = 0.8391
Glucose	93.42 (11.61)	92.50 (10.27)	*p* = 0.9079
Serum bicarb	29.00 (1.95)	27.75 (1.54)	*p* = 1.5792

†Indicates variable is outside of optimal range and ‡indicates outside reference range.

Secondary Outcomes: Figure 3 presents mean results from each participant for gait speed, dynamic balance, freezing of gait, and other functional motor tasks. Clinical improvements were assessed by MCID for the total UPDRS, UPDRS Part III and IV, miniBEST, FoG, and PDQ-39 (Table 2).

## Discussion

To our knowledge, this mixed-methods exploratory pilot study is the first to test a modified, low dairy KD on people with PD. Results demonstrated a modified, low dairy KD was feasible and acceptable for our study sample. As expected, results demonstrated this study’s participants found this intervention tolerable and were highly motivated since this was a participant-initiated study. All predetermined criteria for feasibility were met. Attrition was very low, reported adherence to dietary intake was > 75%, and the recommended daily intake of 80% fat, 15% protein, and 5% carbohydrates was largely followed by participants. When participants ate less fat than recommended, this was replaced with protein rather than carbohydrate content, which may have contributed to the weight loss noted during this study. Urinalysis BHB levels indicated that most participants maintained a ‘nutritional level’ of ketosis by Week 4, with BHB levels reaching at least 3 mM/L.

Predetermined acceptability criteria were met. PDQ-39 scores indicated all participants maintained or improved quality of life. Most subjects experienced no change in constipation, with one subject reporting improvement, and four reporting a slight worsening. It is difficult to determine whether changes in self-reported constipation scores can be solely attributed to the KD, as constipation in PD can be impacted by numerous factors such as natural disease progression (21), stress (22), and microbiome composition (23). Additionally, the pre-study questionnaire asked subjects (*n* = 8) about the number and frequency of bowel movements. Three subjects reported 2 movements per day, while the other 5 subjects had one movement every other day and 5 of the subjects took a stool softener or supplement to help with constipation. It is interesting to note the range of what subjects felt was “normal” for bowel movements, as this may have implications for how constipation was reported in the UPDRS.

Predetermined safety criteria were also met, including subject’s baseline concerns regarding constipation, weight loss and blood lipid concentrations. While all participants experienced some weight loss by week 12, none lost more than 10% of their baseline weight, thus meeting our safety criteria for this time period. Additionally, while neither HDL nor triglycerides changed, both average total cholesterol and average LDL showed a non-significant increase by Week 12 (Figure 2C). Some individuals in this study had a more significant increase in cholesterol. If people are consuming a KD for a longer period, cholesterol should be monitored. Increased levels of total cholesterol and LDL are associated with a reduced risk of PD but increased risk of cardiovascular disease (24), underscoring the importance of monitoring blood lipids.

Although not statistically significant, these subjects had elevated eosinophil and basophil counts not reported in other studies (Figure 2D), but which showed KD alone can increase T cells and natural killer cells (25). It is possible these elevated immune cells were caused by seasonal allergies, as the study was conducted in the spring when pollen counts are high (26).

Regarding the secondary outcomes of this study, several improvements in quality of life and functional movement were noted. To avoid relying on statistical differences that do not involve a clear clinical impact, the concept of minimal clinically important differences (MCID) was employed (27) as is commonly used in PD drug trials and observational studies (28–30). MCID were identified in 8 of the 12 participants in motor function, quality of life, dynamic balance, and self-reported freezing behaviors. On average, the cohort’s UPDRS scores for non-motor experience of daily living (Part I), motor experience and activities of daily living (Part II), and motor examination (Part III) all improved by Week 12. The greatest improvement was seen in Activities of Daily Living (section 2) and Cognition (section 6) of the PDQ-39; however, these were not statistically significant. No significant changes were observed in Mobility, Emotional Well-being, Stigma, and Communication. There was no change in the cohort’s mean for motor complications (UPDRS Part IV).

To our knowledge, five other small clinical trials have investigated the effects of the KD on PD. (5, 31–35) Consistent with our study results, these studies suggest non-motor symptoms may be more likely to improve than motor symptoms when utilizing a KD, at least in the short-term. One randomized trial assigned patients with PD (*n* = 47) to either an 8-week KD or low-fat diet (31) and reported a greater improvement in nonmotor symptoms with the KD, although both groups improved overall motor and nonmotor symptoms. The other four trials, like ours, are single arm trials. Krikorian et al. (32) followed 14 patients with PD-associated mild cognitive impairment during an 8-week KD and found an improvement in lexical access and memory, and a nonsignificant trend toward reduced memory interference (32). Another study used a 12-week KD in patients with PD (*n* = 16) and reported improvements in Part I of the UPDRS and anxiety scores, with no change in motor function (33). The smallest study, a 28-day KD in five patients with PD, reported improved UPDRS scores (5). Finally, Choi et al. (34) utilized a 3-week KD plus medium-chain triglyceride supplementation in 15 patients with PD. They found the diet was feasible and acceptable, and reported a reduced non-motor symptom severity score, with no change in UPDRS, 3-back, and rs-EEG results. As non-motor symptoms are affected by changes in neurotransmitter and inflammation signaling, it is possible they exhibit more plasticity than symptoms resulting from the loss of dopaminergic cells in the substantia nigra that control movement, muscle control, and balance. Dopaminergic decline or restoration of function of injured neurons in PD is not generally considered possible, although mechanistically a KD may favorably impact mechanisms of neurotoxicity and slow disease progression (36).

Several biological mechanisms of a KD may be relevant to PD therapy, including mitochondrial energy production, immunomodulation, reduced circulating insulin and glucose, and altered gut microbiota. A subgroup of patients with PD have been found to have a defect in cellular energy production specifically related to respiratory chain complex I, which requires glucose as a substrate. A KD switches primary energy consumption from glucose to ketones, bypassing the need to utilize the defective complex I and instead primarily utilizing complex II, which may help restore appropriate mitochondrial function and energy production (37). Several murine models showed improved mitochondrial function, increased ATP production, and protection of dopaminergic neurons when KD or ketogenic supplements were utilized after a complex I inhibitor (4, 5, 38–41). These improvements in energy synthesis and protection of dopamine production may be influenced by the activation of the Nrf2-ARE pathway. The KD induces an initial mild oxidative stress, stimulating innate antioxidant responses through Nrf2-ARE signaling. Ultimately, this leads to an overall reduction of oxidative stress (41–44).

Additionally, a KD may modulate the immune system and result in reduced inflammation. A decrease in pro-inflammatory cytokines was observed in preliminary human studies (41, 45), with a decrease in both proinflammatory cytokines and microglia activation noted in murine models (4, 42, 45–47). This combination of reduced oxidative stress, rescued respiratory chain complexes, and decreased inflammation and inflammatory cytokine reduction improved spatial learning and memory, cognitive function, and motor control in KD mouse models (5, 40, 43–45, 47–49).

Since insulin resistance and diabetes mellitus are risk factors for PD (50) and are associated with disease progression and severity, a KD’s significantly reduced carbohydrate consumption may result in improved disease outcomes. Clinical and *in vivo* PD studies using repurposed anti-diabetic medications report mixed results but suggest an overall potential modest benefit (50) in motor and cognitive symptoms with GLP-1 receptor agonists taken for at least 12 months (51). By limiting dietary carbohydrate intake and increasing fat and protein consumption, ketone bodies become the primary source of ATP for tissues, including the brain. Not only could the decrease in circulating glucose potentially alleviate neuronal glucotoxicity, but hepatically produced BHB (one of the two primary endogenous ketones derived from fats) can cross the blood–brain barrier and reduce hippocampal neuron death (4).

Finally, a KD may also modify the gut microbiota due to the significant shift in dietary macronutrient intake. Gastrointestinal symptoms often present years before motor symptoms in PD and evidence suggests gut microbes play a role in PD pathogenesis via the microbiota-gut-brain axis (52). Several studies highlight altered gut microbial populations in PD patients, which may be positively associated with more severe postural instability and gait difficulty (53, 54). Some researchers have recently proposed the potential origination of pathogenic alpha-synuclein proteins in the gastrointestinal tract (54), and transfer to the central nervous system via trans-synaptic cell-to-cell transmission in the vagal nerve. Although this is currently theoretical, the deposition of alpha-synuclein aggregations like those found in the brain have been found in the enteric nervous system.

Our decision to modify the KD by reducing dairy was based on evidence suggesting consumption of higher amounts of certain animal dairy products may increase risk and disease progression in PD (55–57). A meta-analysis of epidemiological data from the US, Finland, and Greece, found a positive, dose–response association between dairy consumption (especially milk and cheese) and the incidence of PD in men (55). Subsequent studies, however, have shown less (57) to no association (58), associations only with certain types of dairy (e.g., milk) (59), or differing associations by fat-content of milk such as positive association with low-fat milk (58) and inverse association with high-fat milk (58). For the purposes of this study, reduction of dairy intake to minimal levels was deemed to be a reasonable modification. Future studies investigating modified KD for individuals with PD should consider subcategorization factors such as: sex, location (pesticide (60) use in different countries/regions), type (fermented vs. non-fermented dairy products), and content (e.g., high fat, low sugar dairy products).

### Strengths and limitations

One limitation of this study was the small sample size of 12 participants. As a participant initiated this study, these subjects were potentially more motivated to adhere to the study and dietary requirements compared to other participant pools.

This is one of the longest clinical studies performed to date on a group with PD utilizing a KD. While this is a longer study duration, it may not be long enough to fully elucidate biological mechanism changes and outcomes associated with a KD in PD. Therefore, longer follow-up times are recommended for future investigations. This study also provided participant education regarding meals as opposed to providing participants with meals. Although this did not allow for monitoring of diet adherence through traditional dietary research methods, this participant education provided a means to increase self-efficacy in disease symptom management. Alternatively, monitoring of self-reported macronutrient levels and BHB from objective peripheral blood analysis provided reasonable confidence in dietary adherence. Due to the high cost of providing participants with meals, the alternative strategy of patient education may more accurately approximate real-world conditions, especially in larger studies of longer duration.

Lastly, while previous research has suggested KD adherence may have a beneficial impact on the gut microbiome as measured by inflammatory and immune (e.g., cytokine) biomarkers, these were not measured in this study. Future studies are needed to quantify microbiome, inflammatory, and immune measures in participants with PD adhering to a KD.

## Conclusion

This exploratory study investigated a modified KD limiting animal dairy products for 3 months in 12 individuals with PD. Initial results were promising, showing this modified KD is acceptable and feasible for individuals with PD. Clinical improvements were also found in total UPDRS, UPDRS Part III, miniBEST, Freezing of Gait, and quality of life, without an increase in constipation or significant weight loss, both of which are concerns in PD. This study shows the modified KD is safe for this duration, may have similar benefits to a traditional KD, and may potentially reduce the risk of symptom exacerbation associated with traditional KD by limiting consumption of animal dairy products. To further elucidate the beneficial biological mechanisms of a KD in individuals with PD, future studies with larger cohorts and longer follow-up times are needed to objectively measure changes in the gut microbiome, circulating metabolic markers, and immunomodulation.

## Data Availability

The raw data supporting the conclusions of this article will be made available by the authors, without undue reservation.
